# The Role of Resolvins, Protectins and Marensins in Non-Alcoholic Fatty Liver Disease (NAFLD)

**DOI:** 10.3390/biom11070937

**Published:** 2021-06-24

**Authors:** Dominika Maciejewska-Markiewicz, Ewa Stachowska, Viktoria Hawryłkowicz, Laura Stachowska, Piotr Prowans

**Affiliations:** 1 Department of Human Nutrition and Metabolomics, Pomeranian Medical University, 70-204 Szczecin, Poland; ewast@pum.edu.pl (E.S.); vik.hawrylkowicz@gmail.com (V.H.); l.stachowskaa@gmail.com (L.S.); 2 Clinic of Plastic, Endocrine and General Surgery, Pomeranian Medical University in Szczecin, 72-009 Police, Poland; pprowans@wp.pl

**Keywords:** NAFLD, n-3, PUFA, resolvins, protectins, marensins

## Abstract

Increased triacylglycerols’ (TAG) synthesis, insulin resistance, and prolonged liver lipid storage might lead to the development of non-alcoholic fatty liver disease (NAFLD). Global prevalence of NAFLD has been estimated to be around 25%, with gradual elevation of this ratio along with the increased content of adipose tissue in a body. The initial stages of NAFLD may be reversible, but the exposition to pathological factors should be limited. As dietary factors greatly influence various disease development, scientists try to find dietary components, helping to alleviate the steatosis. These components include n-3 polyunsaturated (PUFA) fatty acids, especially eicosapentaenoic acid (EPA) and docosahexaenoic acids (DHA). This review focused on the role of resolvins, protectins and merensins in NAFLD.

## 1. Introduction

Increased triacylglycerols’ (TAG) synthesis, insulin resistance, and prolonged liver lipid storage might lead to the development of non-alcoholic fatty liver disease (NAFLD) [1]. NAFLD is defined as lipids’ accumulation in 5% of hepatocytes or fat storage in at least 5% of liver weight [2]. Global prevalence of NAFLD has been estimated to be around 25%, with gradual elevation of this ratio along with the increased content of adipose tissue in a human body [3]. It was estimated that as much as 90% of morbidly obese patients will develop different stages of NAFLD [4]. The most important factors in the etiology of NAFLD include poor eating habits with excessive caloric intake and insufficient physical activity. It has been well documented that diets containing high amounts of simple carbohydrates and saturated fatty acids promote steatosis in the liver [5]. The initial stages of NAFLD may be reversible, but the exposition to pathological factors should be limited. As dietary factors greatly influence various disease phenotypes, scientists try to find dietary components helping to reduce the steatosis. These components include n-3 polyunsaturated (PUFA) fatty acids, especially eicosapentaenoic acid (EPA) and docosahexaenoic acids (DHA) [6]. This review explains mechanisms in which active EPA and DHA derivatives affect liver metabolism and NAFLD phenotype. Furthermore, it focused on the impact of resolvins, protectins, and marensins in liver metabolism and NAFLD pathophysiology, all of which were not discussed in existing reviews.

## 2. Dietary Trends of N-3 Fatty Acids’ Intake

According to a 1990–2010 analysis [7], global mean consumption of PUFA n-3 from seafood, the main source of EPA and DHA, was 163 mg/day, with wild variation between countries (from 5 to 3886 mg/day). Only in 45 out of 187 countries analyzed were mean intakes ≥250 mg/day, which is in line with current guidelines. The other study demonstrated that, in almost 100 countries, the consumption of PUFA n-3 consumption was very low and did not exceed 100 mg/day [8]. Givens et al. reported that current intake of EPA and DHA in Europe ranges from 50 to 344 mg/day [7]. Howe at al. revealed that in persons living in Australia the intake was estimated on 143 mg/day [9].

## 3. N-3 PUFA Supplementation in NAFLD

In 2016, Guo et al. [10] conducted a meta-analysis of randomized controlled trials (RCTs), which aimed to determine the effectiveness of n-3 PUFA supplementation in NAFLD. The study included 11 RCTs and 10 case- control studies. N-3 PUFA supplementation significantly improved alanine aminotransferase (ALT) concentration (−7.53 U/L; 95% CI: −9.98, −5.08; *p* < 0.001). Four trials explored the impact of n-3 PUFA supplementation on liver fat content and the pooled synthesis showed that the mean difference in this parameter in persons receiving the supplementation compared to controls was −5.11% (95% CI: −10.24, 0.02%; *p* = 0.051). Consequently, the authors were able to prove that n-3 PUFA supplementation significantly reduces the ALT, aspartate aminotransferase (AST), and TAG concentrations and, marginally, liver fat content. The study provides substantial evidence that n-3 PUFA supplementation, especially DHA, has a favorable effect in treatment of NAFLD [10].

A meta-analysis conducted by Yan et al. [11], which included 18 studies with a total number of 1424 patients and utilized the fixed effect model, found a significant improvement in liver fat content (RR: 1.56; 95% CI: 1.23 to 1.97, seven studies included), ALT (SMD = −0.50; 95% CI: −0.88 to −0.11, 14 studies included), AST (SMD = −0.54; 95% CI: −1.04 to −0.05, 12 studies included), g-glutamyl transferase (GGT, SMD = −0.48; 95% CI: −0.64 to −0.31, eight studies included), TAG (SMD = −0.47; 95% CI: −0.76 to −0.19; 16 studies included), insulin resistance (HOMA-IR, WMD = −0.4; 95% CI: −0.58 to −0.22; eight studies included), and fasting glucose (SMD = −0.25; 95% CI: −0.43 to −0.06; seven studies included) in persons receiving n-3 PUFA. The results indicated that n-3 PUFA supplementation may improve metabolic and cardiovascular risk factors and surrogate markers for NAFLD progression. However, there was significant interstudy heterogeneity, although a subgroup and meta-regression analyses showed no significantly clear methodological discrepancy [11].

The recent meta-analysis conducted in 2020 by Lee at al. [12], comprising 22 RCTs with 1366 participants, confirmed previous results. The meta evidence was that n-3 PUFA supplementation significantly improves the levels of TAG, total cholesterol, high-density lipoprotein (HDL), and body mass index (BMI), with pooled mean difference and 95% CIs between supplemented persons and controls as follows: −28.57 (−40.81 to −16.33), −7.82 (−14.86 to −0.79), 3.55 (1.38 to 5.73) and −0.46 (−0.84 to −0.08), respectively [12].

Recent research undoubtedly demonstrated that supplementation of n-3 PUFA can support the steatosis reduction and significantly improves key biochemical characteristics of NAFLD. The most popular n-3 source in RCTs included in the abovementioned syntheses was fish oil containing high concentrations of EPA and DHA. Studies underline that these components stand for high biological activity of fish oil [6].

## 4. EPA, DPA and DHA Derivatives

Provided with a diet, α-linolenic acid (ALA) and linoleic acid (LA) undergo changes, catalyzed by many enzymes that extend their structure (elongases) and form double bonds (desaturases). Metabolic changes of ALA and LA take place in the endoplasmic reticulum [13]. As a result of the action of these enzymes (Δ5-, Δ6-desaturases and elongases), ALA forms EPA (C20: 5 n-3), DPA (C22: 5 n-3) and DHA (C22: 6 n-3). The extent of ALA to its metabolites’ conversion varies in terms of gender. For example, in men, ALA to EPA conversion ranges from 6 up to 7.9%, ALA to DPA is approximately 6%, and ALA to DHA frequency does not exceed 1% with a range between 0 and 3.8%. Meanwhile, in women, these percentages are up to 21.1% regarding conversion to EPA, 5.9% to DPA, and 9.2% to DHA [14]. High ALA-to-DHA-conversion ratio in women results from higher demand on DHA supply during pregnancy and lactation [15]. It has also been shown that about 9% of DHA from the diet can be converted back into EPA as a result of DHA β-oxidation [16]. ALA conversion to long-chain derivatives and, consequently, their levels in plasma and phospholipids of red blood cells depends also on the polymorphism of the FADS1 and FADS2, genes coding Δ5- and Δ6-desaturase proteins [17].

The functional relationship between n-3 and n-6 PUFA pathways of their metabolic transformations involves competition for the substrate. The predominance of LA in the diet inhibits the synthesis of EPA and DHA, with the increased synthesis of arachidonic acid (ARA, n-6) [18]. Improper balance between n-3 and n-6 in the diet may result in the disturbance of the physiological balance [19]. The enzymatic competition involves also lipoxygenases (LOX) and cyclooxygenases (COX) and other enzymes responsible for the PUFA transformation to cytokine mediators. DHA and DPA can be converted into anti-inflammatory and organ-protective components, such as D- series of resolvins, protectins, and maresins [20]. EPA, dependent of metabolic factors, is converted to both anti-inflammatory (E- series of resolvins) and inflammatory mediators (prostaglandins, tromboxanes, leukotrienes and hydroxy acids) [21]. The products of n-3 PUFA conversion are provided in Table 1 [22,23,24,25,26,27].

## 5. Resolvins

Resolvins are cytokines of an anti-inflammatory nature produced during EPA, DPA and DHA metabolism. E- series of resolvins are produced by oxygenation of EPA, a process catalyzed by ACA-COX-2, resulting in 18-HpEPE formation [28]. The next step requires the reduction of 18-HpEPE to 18-HEPE and oxygenation by 5-LOX. This hydroperoxide metabolite is converted via a hydrolyzation pathway to RvE1. The reduction of hydroperoxide by a peroxidase can generate RvE2 [29]. D-series of resolvins are produced in two oxygenation steps. The first step is mediated by 15-LOX and results in the formation of 17-HpDHA, which is then quickly reduced to 17-HDHA. The next oxygenation step requires 5-LOX and leads to the formation of a peroxide intermediate that is reduced to RvD5 and further hydrolyzed to RvD1 and RvD2 [27]. At the same time, the oxygenation by 5-LOX at the C-4 carbon position generates RvD3, RvD4 and RvD6 [30]. DPA is a precursor of two series of resolvins: D- and 13-series with a DPA core. Resolvin D-series from DPA are produced by the same enzymes as DHA. The 13-series of resolvins are produced by the oxygenation with COX-2 to 13-HDPA and S-nitrosylation [31].

COX-2 is widely known to mediate prostaglandin production. However, it can be acetylated in the presence of aspirin or other non-steroidal drugs. ACA-COX-2 does not catalyze prostaglandins’ production but mediates the resolvins’ and protectins’ formations [32]. Simon et al. revealed that daily aspirin use was associated with less severe histological features of NAFLD and NASH and lower risk for progression to advanced fibrosis with time [33].

In vitro studies revealed that RvD1 reduced apoptosis and tunicamycin-induced TAG accumulation through c-Jun N-terminal kinase (JNK) pathway in HepG2 cells. Furthermore, the resolvin significantly decreased TAG accumulation and SREBP-1 expression [34]. Animal studies proved that resolvins have a great influence on NAFLD course. Rodriguez et al. showed that RvE1 (administered in a regimen of 100 ng/body weight, twice weekly for four weeks) suppressed fibrosis in Sprague–Dawley rats, which received diethylnitrosamine (70 mg/mg body weight intraperitoneally) once a week. RvE1 intake normalized albumin, ALT, and lactate dehydrogenase (LDH) levels and decreased the histological distortion, inflammatory infiltration, necrotic areas, and microsteatosis [35]. González-Périz conducted research, in which n-3 PUFA was administered to ob/ob mice, being an animal model of fatty liver disease. The results proved that the expression of genes involved in insulin and glucose metabolism (namely PPARγ and GLUT-2/GLUT-4) and insulin receptor (IRS-1/IRS-2) were up-regulated. Further analysis showed that PUFA metabolites decreased the formation of pro-inflammatory eicosanoids originated from n-6 PUFA and enhanced the formation of resolvins and protectins. Furthermore, RvE1 and PD1 limited the insulin-sensitizing effects and increased adiponectin expression similarly to rosiglitazone (antidiabetic drugs) [36]. Rius et al. tested the ability of RvD1 to improve the metabolic parameters initiated by caloric restriction in obese mice with non-alcoholic steatohepatitis (NASH). In order to reduce body weight and fat content, mice underwent 40% calorie restriction diet and were administered with RvD1 (300 ng/day) or placebo. In mice administered with the intervention product, adiponectin expression at mRNA and protein levels increased and liver macrophage infiltration was inhibited. Moreover, RvD1 induced macrophages’ transformation from M1- to M2-like anti-inflammatory phenotype and initiated macrophage immune response. In the liver tissue, the resolving supply decreased hypoxia-induced expression of COX-2, IL-1β and IL-6 [37]. Similar results were provided by Hellmann et al., who evaluated whether RvD1 (2 μg/kg) administration improves insulin sensitivity by diminishing chronic inflammation associated with obesity. The study results provided evidence that RvD1 improved blood fasting glucose, increased adiponectin production and simultaneously decreased the expression of IL-6 in adipose tissue. Moreover, macrophages’ F4/80 + CD11c + structure formation was reduced by >50% in adipose tissue [38]. Pal et al. investigated the effects of 4-day RvE1 administration in C57BL/6J mice and found that such intervention diminished hyperinsulinemia and hyperglycemia [39].

## 6. Protectins

Protectins are anti-inflammatory molecules produced from DHA and DPA. A first compound detected from the protectin family was PD1 [40]. It is produced by oxygenation of DHA/DPA through a pathway activated by 15-LOX [30]. The 15-LOX generates 17-HpDHA, which is rapidly converted to a 16, 17-epoxide-containing molecule after the epoxidation to PD1 [41]. Protectin DX (PDX), an isomer of protectin/neuroprotectin D1 derived from DHA, enhances the palmitate-induced TAG accumulation through the regulation of SREBP1 pathway. When HepG2 cells were treated with PDX, the suppression of endoplasmic reticulum stress via AMPK-induced ORP150 expression was found. Additionally, reduced hepatic steatosis induced by a high-fat diet was detected [42]. González-Périz et al. examined the effect of n-3 PUFA supplementation (6% of the lipid in the diet came from by n-3 PUFA) in ob/ob mice. A mass spectrometry lipidomic analysis showed that n-3 PUFA reduced the formation of pro-inflammatory eicosanoids derived from n-6 PUFA and increased the formation of resolvins and protectins. The study provided evidence that RvE1 and PD1 possess the insulin-sensitizing and antisteatotic effects similarly to the antidiabetic drug rosiglitazone. The study confirmed that PDX-associated IL-6 release promotes hormone-dependent suppression of hepatic glucose [36].

There is still limited information about role of protectins in NAFLD. Maciejewska et al. found that, during NAFLD progression, the concentration of protectins’ D1 does not change significantly [43]. Protectins have a great impact on macrophage polarization (a process by which macrophages produce distinct functional phenotypes as a reaction to a specific microenvironment) [44], which is associated with the pro-inflammatory state in adipose and liver tissues. Macrophage polarization to M1 phenotype and increased ratio of M1/M2 induce proinflammatory signals and make the adipocytokines from adipose tissue to be released [45]. Negative macrophage polarization and increased release of inflammatory cytokines are very important factors in NAFLD pathogenesis and progression [46].

## 7. Maresins

Maresins are DHA- and DPA-derived molecules produced by macrophages [47]. Maresins are biosynthesized via lipoxygenation by placing molecular oxygen at the carbon-14 position. The biosynthesis of maresins is initiated by 12-LOX and involves DHA and DPA oxygenation. Afterwards, 14-hydroperoxy-intermediate is epoxidated and converted to 13, 14-epoxy-maresin. Moreover, 13, 14-epoxide intermediates inhibit the 12-LOX conversion of eicosatetraenoic acid. It is considered that 13, 14-epoxide intermediates might have a positive influence on the pathway of maresin biosynthesis and boost anti-inflammatory effect [48].

Maresins are able to decrease the synthesis of proinflammatory cytokines, namely, TNF-α, IL-1β and IL-6. Moreover, maresins inhibit neutrophil infiltration, restrict the further recruitment of polymorphonuclear leukocytes (PMNs), and excite the nonphlogistic recruitment of mononuclear cells [49]. Maresins might also reduce the inflammation via lowering the production of leukotriene B4 (LTB4) and inhibition of leukotriene A4 hydrolase (LTA4H.) [50].

As a proresolving lipid mediator, MaR1 activates protein kinase C, which results in limited infiltration of neutrophil and lowered levels of chemokine C-X-C motif ligand 1, IL-6 and TNF- α [51]. Viola et al. confirmed that MaR1 prevented atheroprogression in smooth muscle cells by changing macrophage profile, making a reparative phenotype to be originated, and stimulated the synthesis of collagen, enhancing overall healing abilities [52]. In another study, it was proven that MaR1 reduced TNF-α, IL-1β, monocyte chemotactic protein 1 (MCP-1), and the proinflammatory M1 macrophage phenotype marker Cd11c expression and upregulated glucose transporter-4 protein (Glut-4) and adiponectin in diet-induced obese (DIO) mice. MaR1 supply increased adiponectin gene expression and improved the insulin tolerance test, Akt and AMPK phosphorylation, and IL-10 synthesis in ob/ob mice [53]. Maresin 1 may also improve diabetic nephropathy by decreasing fibronectin (FN), NLRP3 inflammasome and TGF-β1 expression in mouse glomerular mesangial cells [54].

Jung et al. tested MaR1 action under hyperlipidemic conditions and noticed that MaR1 reduced the hepatocyte endoplasmic reticulum stress and reduced lipid deposition in the liver. Moreover, MaR1 can increase AMP-induced protein kinase activity, which is associated with increased Ca^2+^—ATPase 2b (SERCA2b) expression in the sarcoendoplasmic reticulum [55]. It was shown that administration of MaR1 increased Serca2b mRNA expression and hepatic AMPK phosphorylation, while ER hepatic stress was reduced in mice administered with a high-fat diet (HFD). In addition, treatment with MaR1 inhibited hepatic lipid synthesis, thus limiting steatosis in the liver of HFD-fed mice [55].

Laiglesia et al. conducted a study in DIO mice. Animals were fed with MaR1 (2–10 μg kg^−1^ i.p., 20 days and 2 μg kg^−1^, i.p., or 50 μg kg^−1^) by oral gavages for 10 days, respectively. Maresin administration reduced liver steatosis via decreasing lipogenic enzymes’ expression (fatty acid synthase (FAS) and stearoyl-CoA desaturase-1) and influenced AMPK activation by inducing autophagy. The intervention also decreased the level of TAG in the liver in mice with obesity-related hepatosteatosis. These reports suggest that MaR1 might be a useful tool in the treatment of NAFLD by reducing hepatocyte lipogenesis induced by stress in the endoplasmic reticulum [56].

Maresin 2 (MaR1), the second member of maresins’ family, namely 13, 14-diHDHA, is also produced via 12-LOX activity [57]. MaR2 plays a role in limiting PMN infiltration, similarly to MaR1. However, there is still limited data on properties of MaR2 in the context of NAFLD.

## 8. Conclusions

**Figure 1:**
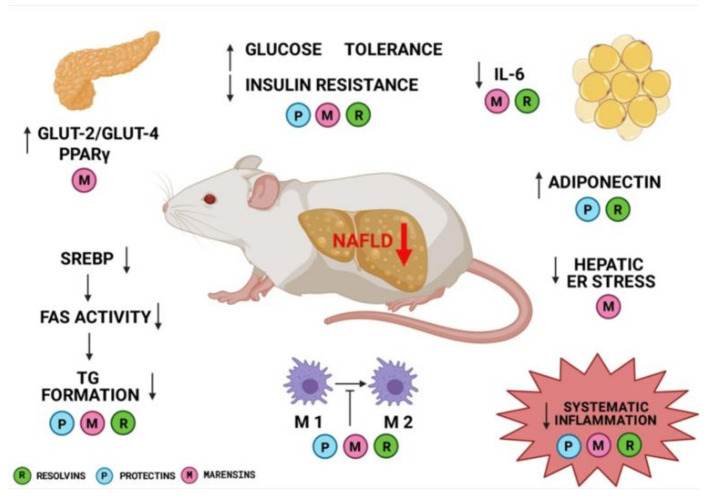
Resolvins’, protectins’ and marensins’ mechanisms of action. Created with BioRender.com (22 April 2021).

N-3 fatty acids and their derivatives have a beneficial effect in many diseases, including NAFLD. Despite the fact that n-3 supplementation supports NAFLD treatment, there is still insufficient information about the role of EPA, DHA and DPA derivatives in preventing the disease progression. In vitro and in vivo studies showed that all anti-inflammatory derivatives of n-3 fatty acids may have a similar mechanism of action, including decrease of inflammation, reduction of lipogenesis in the liver, and improvement of insulin sensitivity (Figure 1). It should be highlighted that the therapeutic implication of resolvins’, marensins’ and protectins’ supply in NAFLD supportive therapy still remains unclear. In recent clinical trials, the supplementation was based on fish oil, n-3, or EPA and DHA supplementation. In the concept of PUFA supplementation, we cannot predict the enzymatic pathways of n-3 derivatives’ productions. However, clinical trials should verify the findings to further consider these compounds as beneficial supplements in NAFLD patients.

## Figures and Tables

**Table 1:** Enzymatic deratives of EPA, DHA and DPA.

EPA	
Enzyme	Derivatives
P 450	20-hydroksyeicosapentaenoic acid (20-HEPE)
P 450/ACA-COX-2	18-hydroperoksyeicosapentaenoic acid 18-HpEPE
ACA-COX-2/5LOX	Resolvin E1, E2 (RvE1, RVE2)
P-450/5-LOX	Resolvin E3 (RvE3)
LOX-5	5-hydroxyeikozapentaenoic acid (5-HEPE)
ACA-COX-2	Leukotoriene A5 (LTA5)
COX-1/2	Leukotiene B5 (LTB5)
	5- hydroksyoxopentaenoic acid (5-oxo-EPA)
	Prostaglandin G3
	Prostacyclins I3
	Tromboxanes 3
**DHA**	
ACA-COX-2, 15-LOX	17-hydroperoksyeicosapentaenoic acid 17-HpDHA
5-LOX	7- hydroxyoxodocosaheksaenoic acid (7-oxo-DHA)
12-LOX	14-hydroperoksyeicosapentaenoic acid 14-HpDHA
ACA-COX-2, 15-LOX	Marensins 1,2 (MaR1, MaR2)
ACA-COX2, 15/5-LOX	Protectins 1 (PD1)
	Resolvins D1–6 (RvD1, RvD2, RvD3, RvD4, RvD5, RvD6)
**DPA**	
15/5-LOX	Resolvins D1,D2,D5 (RvD1, RvD2, RvD5)
COX-2	Resolvins 13-series (RvT1, RvT2, RvT3, RvT4)
ACA-COX-2	17-hydroperoksydocosapentaenoic acid 1(7-HpDPA)
15-LOX	Protectins 1,2 (PD1, PD2)
12-LOX	Marensins 1,2 (MaR1, MaR2)
